# Storage Life of Particle-Filled Polymer Composites Considering Aging Effects

**DOI:** 10.3390/polym16131893

**Published:** 2024-07-02

**Authors:** Yujiao Zhang, Congli Fang, Huizhen Wang, Minghua Zhang, Tao Shen, Jianke Du

**Affiliations:** Zhejiang-Italy Joint Lab for Smart Materials and Advanced Structures, School of Mechanical Engineering and Mechanics, Ningbo University, Ningbo 315211, China; 2111081032@nbu.edu.cn (Y.Z.); 2011081025@nbu.edu.cn (C.F.); wwwhz777@163.com (H.W.); zhangminghua@nbu.edu.cn (M.Z.)

**Keywords:** particle-filled polymer composites, aging, crosslink density, constitutive model

## Abstract

This study investigates the storage life of particle-filled polymer composites (PFPCs) under the influence of aging effects. High-temperature accelerated aging tests were conducted at 60 °C, 70 °C, and 80 °C for various days to analyze the impact of aging time and temperature on the mechanical behavior of the materials. A predictive model for crosslink density was established using the Arrhenius equation, and the relationship between crosslink density and relaxation modulus was determined based on polymer physics theory. On this basis, a viscoelastic constitutive model that incorporates aging effects was developed. Structural analyses of a PFPC column with a length of 2.3 m and outer diameter of 1.8 m were performed using the UMAT subroutine in ABAQUS. Subsequently, a safety margin assessment method based on dewetting strain was employed to predict the storage life of the PFPC column. The results indicate that the aging viscoelastic constitutive model effectively characterizes the hardening effects caused by aging in the composites during storage. The storage life for the PFPC column considering aging effects decreases from 22 years to 19 years compared to models that ignore such effects. This approach provides a reference for estimating the storage life of PFPC columns considering aging effects.

## 1. Introduction

Particle-filled polymer composites (PFPCs), thanks to their excellent mechanical properties, are widely used in industries such as machinery, aerospace, and medical devices [1,2,3]. PFPCs with energetic particles, such as the solid fuels used in aerospace applications, are usually required for long-term storage [4,5]. During storage, they are prone to thermal–oxidative aging [6,7,8]. The microstructure will change during aging, which is the critical factor for the degradation of macroscopic mechanical properties. Thus, based on both safety and cost-effectiveness considerations, it is of great significance to explore the aging mechanism and establish an effective model to predict the storage life of PFPCs.

In research focused on predicting the storage life of PFPCs, the mechanical properties are initially forecasted through aging experiments, with subsequent calculations of the mechanical responses using a viscoelastic constitutive model. Throughout the prolonged storage period, aging affects the structural responses through constitutive relationships. Thus, studies on viscoelastic constitutive equations related to aging have garnered extensive attention [9,10]. Yang et al. [11] investigated the time-dependent mechanical properties of composite materials and developed a viscoelastic constitutive model that incorporates aging effects. Similarly, Rahmani et al. [12] constructed a coupled oxidative aging–viscoelastic constitutive relationship and predicted the viscoelastic response of aged asphalt concrete materials. Furthermore, Ozer et al. [13] established an aging viscoelastic constitutive model by integrating aging parameters into their analysis. Despite the extensive research on aging viscoelastic constitutive models, it remains a challenging task to correlate aging characteristics at various scales so as to establish an effective aging constitutive model.

To more accurately predict the aging mechanical behavior of PFPCs, researchers are dedicated to uncovering the intrinsic mechanisms during the aging process and enhancing the understanding of their aging characteristics. Extensive studies have shown that oxidative crosslinking and degradation of the adhesive polymer network, as well as the migration and volatilization of components, are critical aging factors [14,15,16,17]. Crosslink density is a vital indicator of the extent of material aging. Changes in this parameter directly impact the macroscopic mechanical properties of the material, such as strength, elongation, and modulus [18,19,20,21]. Consequently, the development of corresponding viscoelastic constitutive models can be guided by predicting the aging characteristics through crosslink density [22,23]. This can provide significant guidance for studying the structural response of PFPCs.

In this study, thermal accelerated aging tests on PFPC specimens are first performed, followed by tests of crosslinking density, relaxation modulus, and dewetting strain. Subsequently, a viscoelastic constitutive model incorporating aging factors is established. By utilizing the subroutine functions of ABAQUS, this constitutive model is implemented to analyze the structural response of the PFPC column. Finally, a safety margin assessment method based on dewetting strain is employed to estimate the storage life of the column. This assessment method provides a reference for the storage and life extension management of PFPCs.

## 2. Materials and Methods

**Figure 1:**
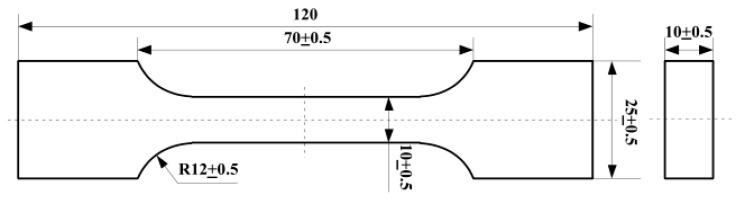
Diagram of specimen (unit: mm).

This study employs PFPC specimens for all experimental procedures. The matrix of the specimens is composed of hydroxyl-terminated polybutadiene and toluene diisocyanate, while the fillers include ammonium perchlorate particles. After completing the composition as described, the mixture is stirred and cast under vacuum conditions, then cured into cuboid specimens for subsequent processing. To ensure even stress distribution during testing, the cured cuboid specimens are fashioned into dumbbell-shaped specimens. The cross-sectional dimensions are 10 × 10 mm, as shown in Figure 1.

The study employs thermal accelerated aging tests to investigate changes in the mechanical properties of PFPC during the aging process. When selecting temperature points for artificial accelerated aging experiments, the lowest aging temperature should be close to the actual storage temperature of the propellant, but not too close, to avoid excessively extending the testing time. The highest aging temperature should be as high as possible to achieve a strong accelerating effect while ensuring safety and not altering the failure mechanism. It is recommended to use three to five temperature points to balance data accuracy and experimental cost. Based on these principles, this study selected three aging temperatures: 60, 70, and 80 °C. The specimens are sealed in aluminum foil bags and placed in an oven set at temperatures of 60 °C, 70 °C, and 80 °C for aging. At intervals, some specimens are removed for relaxation tests, uniaxial tensile tests, and equilibrium swelling tests, with specific sampling times, as shown in Table 1 [24].

Under external forces, PFPCs exhibit viscoelastic characteristics. To obtain the viscoelastic parameters of PFPCs, stress relaxation tests were conducted on both unaged specimens and some aged specimens. The specimens selected at different aging stages are detailed in Table 2 with their respective aging times.

**Figure 2:**
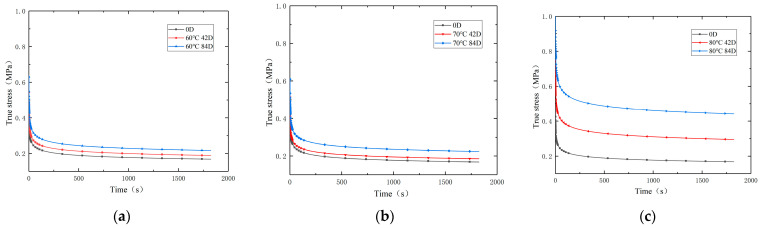
Line chart of the relationship between true stress and relaxation time: (**a**) aged at 60 °C; (**b**) aged at 70 °C; (**c**) aged at 80 °C.

The relaxation tests were carried out at room temperature, with a test temperature of 25 °C and humidity not exceeding 70%. The specific procedure of the test is as follows: First, the specimens, using standard dumbbell-shaped special fixtures, were mounted on a universal testing machine. Then, the specimen was subjected to a constant speed tensile test at a loading rate of 100 mm/min with the loading time set to 2 s. Subsequently, the strain was held constant, and the relaxation time was set to 1800 s. The results of the relaxation test are shown in Figure 2. From the figure, it can be seen that the stress relaxation can be divided into two stages. In the first phase, the stress decreases rapidly with time; in the second phase, the rate of stress reduction gradually slows down and eventually stabilizes.

**Figure 3:**
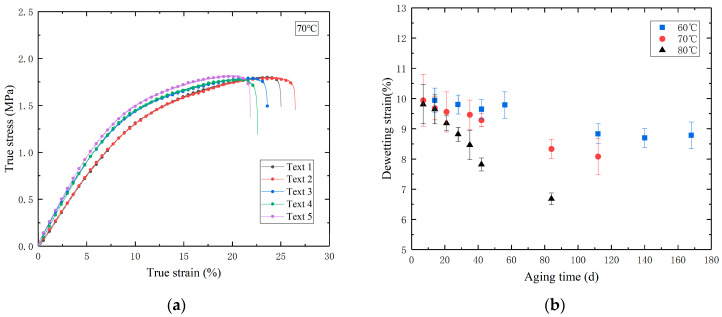
(**a**) Line chart of the relationship between true stress and true strain; (**b**) line chart of the relationship between dewetting strain and aging time during the accelerated aging process.

To investigate the impact of aging on the mechanical properties of PFPCs, uniaxial tensile tests were performed on aged specimens. The test procedure is as follows: The universal testing machine was set to a uniform stretching mode at a rate of 100 mm/min. The test then proceeded by stretching the specimen until it completely fractured, with data recorded throughout. The resulting stress–strain curve is shown in Figure 3a. As the strain increases, the slope of each point on the curve gradually changes. Under the influence of external forces, relative motion occurs between the binder matrix and solid filler particles within the PFPCs. This motion leads to the gradual failure of the interface between the matrix and the filler, eventually causing interface separation. The point on the uniaxial tensile test stress–strain curve where the slope changes most significantly is known as the dewetting point [25,26], and the dewetting strain is shown in Figure 3b. From the figure, it can be observed that under three aging temperatures, the dewetting strain decreases as the aging time increases.

**Figure 4:**
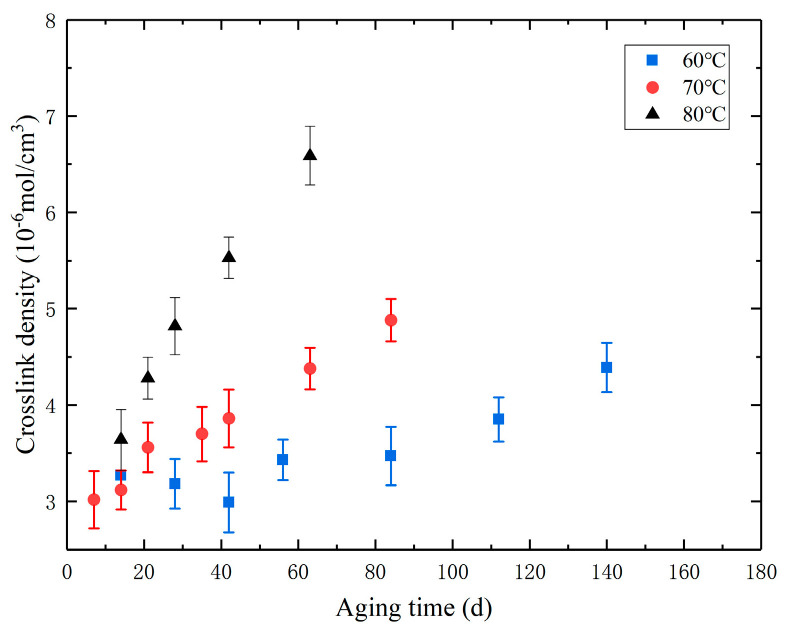
Line chart of the relationship between crosslink density and aging time during the accelerated aging process.

Crosslink density is an important parameter affecting the mechanical properties of materials, and changes in it reflect alterations in the internal structure of the material due to aging. The crosslink density of the aged specimens is determined through equilibrium swelling tests [27]. The specific method for the equilibrium swelling test is as follows: First, the specimens were removed at the sampling set time and were cut into thin circular disk samples with dimensions of Φ20 mm × 2 mm, and their mass was measured. Subsequently, these disks were immersed in toluene for swelling tests; the toluene was replaced every two days to ensure full swelling within seven days. After swelling, the surface solution was removed from the samples, and their mass was measured again. Finally, the crosslink density values were calculated according to Flory–Huggins theory, and the results are shown in Figure 4. The graph indicates that the trend in crosslink density changes similarly across all temperatures. Under various aging temperatures, the crosslink density increases with the aging time.

## 3. Aging Viscoelastic Constitutive Model

### 3.1. Crosslink Density Prediction Model

As storage time extends, under the influence of internal structural instability and external environmental complexities, PFPCs undergo a series of complex aging processes [28]. Studies have shown that during aging, the microstructure of the PFPCs matrix undergoes a series of changes [29]. On the one hand, under thermal effects, reactive oxygen attacks the tertiary carbon atoms in the butanone polyurethane network, leading to chain scission phenomena. On the other hand, crosslinking reactions occur between butanone polyurethane networks, such as reactions between peroxyl or alkyl radicals and C=C double bonds and crosslinks formed between reactive radicals produced in free radical chain termination [30,31]. These chemical changes are significant factors in the aging of PFPCs. In the PFPCs used herein, oxidative crosslinking reactions dominate over chain scission effects [32]. Hence, it is considered the primary aging mechanism for this material.

Therefore, during storage, the change in crosslink density is a key parameter for assessing changes in the network structure. This study constructs a crosslink density prediction model. The model accurately reflects the trend of changes in the crosslink density of PFPCs during the aging process. It provides a quantitative analytical method for the aging behavior of PFPCs.

Before establishing the crosslink density prediction model, two basic assumptions were made. Firstly, it is assumed that in the thermal accelerated aging tests, the aging mechanism of the specimens is consistent with that at room temperature [33]. This implies that, during the storage aging process, the rate of change in crosslink density is only related to the thermal aging temperature while not directly to other potential influencing factors.

Secondly, it is presumed that during the thermal accelerated aging process, the rate of change in crosslink density of the specimens follows the Arrhenius equation. The Arrhenius equation is a fundamental equation in chemical reaction kinetics and a commonly used semi-empirical formula for studying the aging characteristics of materials [34]. It describes the relationship between the reaction rate constant and temperature. This study uses this equation to simulate the rate of change in crosslink density, providing an important theoretical framework for understanding and predicting material aging. It is assumed that the crosslink density evolves as:

(1) $$v=v_{0}+kt$$

where *v* represents the crosslink density value at a given time, *v*_0_ is the initial value of crosslink density, *k* is the rate constant of change in crosslink density, and *t* is the aging time.

The rate constant of change in crosslink density *k* can be expressed as:

(2) $$k=Z\exp(-\frac{E_{a}}{RT})$$

where *Z* is the pre-exponential factor, *E_a_* represents the apparent activation energy, *R* is the molar gas constant, and *T* is the aging temperature.

**Figure 5:**
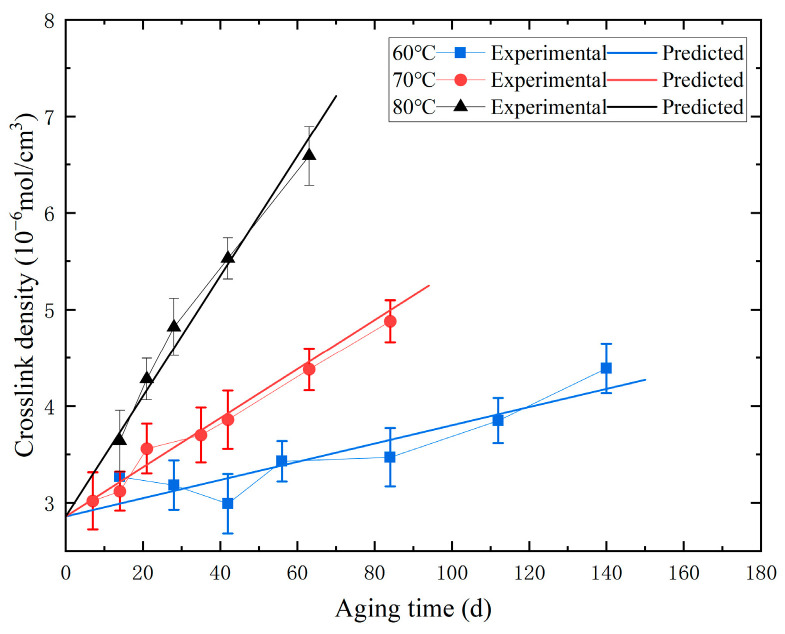
The comparative diagram of experimental/predicted crosslink density.

For the obtained crosslinking density data, the relationship between crosslinking density, aging time, and aging temperature can be fitted using the least squares method. The fitting results show *v*_0_ = 2.86 × 10^ − 6^ mol/cm^3^ and *k* = e^30.73 − 11,800/T^ × 10^−6^ mol/(cm^3^ s) in Equation (1). According to calculations, the R^2^ value of the crosslinking density prediction model is 0.98, which indicates that the model has an acceptable accuracy and reliability. The comparison results between experimental and predicted crosslinking densities are shown in Figure 5. 

### 3.2. Aging Viscoelastic Constitutive Model Based on Crosslink Density

Before the derivation of a viscoelastic constitutive model, it is necessary to establish a quantitative relationship between the aging relaxation modulus and the degree of aging. PFPC is a polymer with a crosslinked network, and there is a certain mathematical relationship between its relaxation modulus and crosslink density. Based on the elastic physical properties of rubber, for an ideal crosslinked network, its shear modulus can be expressed as:

(3) G=vRT

where *v* represents the crosslink density of the polymer, *R* is Planck’s constant, and *T* is the temperature.

However, for real crosslinked polymers, their crosslink networks are always incomplete. Flory modified Equation (3), and the shear modulus can be expressed as:

(4) $$G=RT(v-\frac{2\rho }{M})$$

where *M* is the initial molecular weight of the polymer before aging, and ρ is the polymer density.

There is a linear relationship between shear modulus and crosslink density. Therefore, the aging shear modulus can be approximated as:

(5) $$G^{t^{\prime }}(t)=R(v)\times G^{0}(t)+G^{0}(t)$$

where $G^{t^{\prime }}(t)$ represents the shear modulus after aging, $G^{0}(t)$ represents the shear modulus before aging, and R(v) represents the thermal aging characteristic function.

The thermal aging characteristic function of the material is related to the aging temperature and aging time. The constitutive model will be more complex by introducing temperature and time variables into the thermal aging characteristic function. The degree of material aging can be characterized by the crosslink density. Therefore, this study establishes a thermal aging characteristic function based on crosslink density to achieve the purpose of simplifying calculations. Under different aging temperatures and aging times, the relationship between crosslink density and the thermal aging characteristic function is linear. The expression of the thermal aging characteristic function with crosslink density is represented by:

(6) $$R(v)=c\times (v-v_{0})$$

In the text, changes in the Poisson’s ratio due to temperature and time are not considered, meaning that the material’s Poisson’s ratio *μ* is assumed to be constant. The relationships between the shear modulus incorporating aging factors, the bulk modulus incorporating aging factors, and the relaxation modulus incorporating aging factors are shown in Equations (7) and (8).

(7) $$G^{t^{\prime }}\left(t\right)=\frac{E^{t^{\prime }}(t)}{2(1+\mu )}$$

(8) $$K^{t^{\prime }}\left(t\right)=\frac{E^{t^{\prime }}(t)}{3(1-2\mu )}$$

Thus, the relaxation modulus incorporating aging factors can be expressed as:

(9) $$E^{t^{\prime }}(t)=R(v)\times E^{0}(t)+E^{0}(t)$$

where $E^{t^{\prime }}(t)$ is the relaxation modulus after aging, $E^{0}(t)$ is the relaxation modulus before aging, and R(v) is the thermal aging characteristic function.

Due to the introduction of the thermal aging characteristic function, which changes with crosslink density, the impact of aging on the mechanical properties of the material can be reflected by the relaxation modulus $E^{t^{\prime }}(t)$.

This study establishes a viscoelastic constitutive model that incorporates aging factors based on the linear viscoelastic constitutive model. The employed viscoelastic constitutive model is a relaxation-type integral constitutive model that is more convenient for finite element discretization, and its one-dimensional form is as follows:

(10) $$\sigma \left(t\right)=\int_{0}^{t}E(t-\tau )\frac{\partial \varepsilon }{\partial \tau }d\tau$$

where *σ*(*t*) represents stress, *ε* represents strain, and *E*(*t*) represents relaxation modulus.

By ignoring changes in Poisson’s ratio during storage and substituting the post-aging relaxation modulus into Equation (10), we obtain the viscoelastic constitutive model that incorporates aging factors, as follows:

(11) $$\tilde{\sigma }\left(t\right)=\int_{0}^{t}E^{t^{\prime }}(t-\tau )\frac{\partial \varepsilon }{\partial \tau }d\tau$$

A three-dimensional viscoelastic constitutive model is required for the structural analysis of PFPCs. Thus, the viscoelastic constitutive model that includes aging factors needs to be extended from one-dimensional to three-dimensional. When extending the one-dimensional viscoelastic constitutive model of isotropic materials, the stress tensor and strain tensor can be divided into volumetric and deviatoric parts, and these two parts are not coupled. Therefore, the stress can be expressed as:

(12) $$\begin{array}{ll} {\tilde{\sigma }}_{ij}\left(t\right) & ={\tilde{S}}_{ij}(t)+\frac{1}{3}\delta_{ij}{\tilde{\sigma }}_{kk}\left(t\right)\\ & =\int_{0}^{t}2G^{t^{\prime }}(t-\tau )\frac{\partial e_{ij}}{\partial \tau }d\tau +\frac{\delta_{ij}}{3}\int_{0}^{t}3K^{t^{\prime }}(t-\tau )\frac{\partial \varepsilon_{kk}}{\partial \tau }d\tau \end{array}$$

where ${\tilde{S}}_{ij}(t)$ represents the deviatoric stress tensor due to aging, ${\tilde{\sigma }}_{kk}\left(t\right)$ represents the volumetric stress tensor due to aging, and $\delta_{ij}$ represents the Kronecker delta (when *I* = *j*, $\delta_{ij}$ = 1; when *I* ≠ *j*, $\delta_{ij}$ = 0).

To simplify the analysis process, this study assumes that the loading process in the universal testing machine follows a uniform rise mode. Based on the relaxation-type integral viscoelastic constitutive model, the stress responses were further mathematically derived during the uniform stretching phase and relaxation phase.

In relaxation tests, strain can be expressed as follows:

(13) $$\varepsilon \left(t\right)=\left\{ \begin{array}{l} {\dot{\varepsilon }}_{0}t\;\;t<t_{0}\\ \varepsilon_{0}\;\;t>t_{0} \end{array}\right.$$

During the uniform stretching phase, the strain rate remains constant. In the stress relaxation phase, the strain remains constant. Therefore, throughout the test process, the stress response can be represented by Equation (14).

(14) $$\sigma \left(t\right)=\left\{ \begin{array}{l} {\dot{\varepsilon }}_{0}\int_{0}^{t}E(t-\tau )\frac{\partial \varepsilon }{\partial \tau }d\tau \;t<t_{0}\\ {\dot{\varepsilon }}_{0}\int_{0}^{t_{0}}E(t-\tau )\frac{\partial \varepsilon }{\partial \tau }d\tau \;t>t_{0} \end{array}\right.$$

This study uses a 10^th^-order Prony series to characterize the relaxation modulus, as shown in Equation (15).

(15) $$E(t)=E_{\infty }+\sum_{i=1}^{10}E_{i}\exp(-\frac{t}{\tau_{i}})$$

where *E_∞_* is the equilibrium modulus, *E_i_* is the coefficient of the *i*-th term of the Prony series, and $\tau_{i}$ is the relaxation characteristic time of the *i*-th term of the Prony series.

In relaxation tests, the required moments *t*_0_, strain rate ${\dot{\varepsilon }}_{0}$, and the stress values at various moments *σ*(*t*) can be obtained. Using these data inserted into Equation (14), the relaxation modulus of the unaged specimens is fitted, as shown in Table 3.

**Figure 6:**
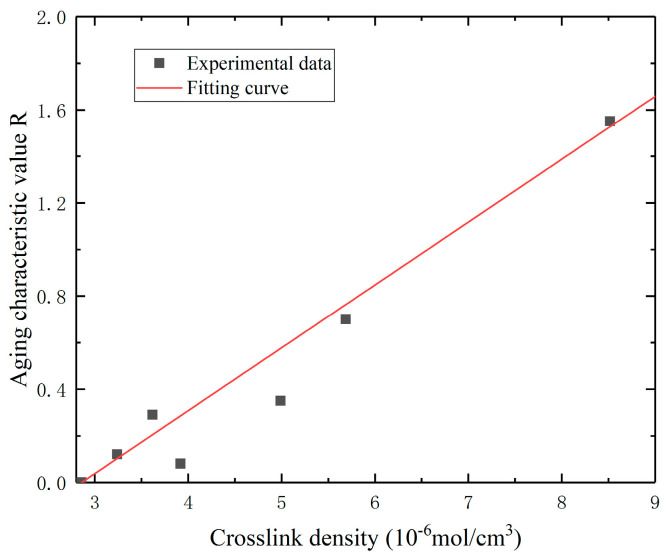
Diagram of crosslink density–aging characteristic value fitting for the specimen.

Based on the uniaxial tensile stress–strain curve data of the aged specimens, the values of the thermal aging characteristic function are calculated. The data on crosslinking density and thermal aging characteristic functions were used to successfully construct a model that describes their relationship. The fitting result shows *c* = 300,000 cm^3^/mol in Equation (6). Through fitting analysis, we obtained a predictive model for the thermal aging characteristic function with an R^2^ value of 0.95. This indicates that the model is accurate and reliable. The fitting results are shown in Figure 6.

### 3.3. Discretization of the Aging Viscoelastic Constitutive Model

During the storage process, PFPCs gradually age and exhibit distinct nonlinear characteristics. To deeply study the mechanical behavior of PFPCs during long-term storage, this study uses ABAQUS 2022 software to model and analyze PFPCs. ABAQUS offers a rich user subroutine interface, facilitating the development of custom subroutines. This study has further developed the aging viscoelastic constitutive through the UMAT subroutine. Considering that the structural response of PFPCs is closely related to the load history, this study employs an incremental method for solving. The main purpose of this method is to determine the relationship between the material stress increment and strain increment within the incremental time step. At the same time, we have calculated the tangent stiffness matrix for further analysis and calculations.

For Equation (12), its incremental form at any incremental step can be expressed as:

(16) $$\Delta {\tilde{\sigma }}_{ij}\left(t\right)=\Delta {\tilde{S}}_{ij}(t)+\frac{1}{3}\delta_{ij}\Delta {\tilde{\sigma }}_{kk}\left(t\right)$$

The increment of the stress deviator tensor is as follows:

(17) $$\begin{array}{ll} \Delta {\tilde{S}}_{ij}\left(t+\Delta t\right) & =2G_{0}^{t+\Delta t}\Delta e_{ij}-\sum_{i=1}^{10}2G_{i}^{t+\Delta t}\frac{\Delta e_{ij}}{\Delta t}\left\{\Delta t-\tau_{i}(1-\exp(-\frac{\Delta t}{\tau_{i}}))\right\}\\ & +\Delta E_{0}^{t+\Delta t}e_{ij}(t)-\sum_{i=1}^{10}2\Delta G_{i}^{t+\Delta t}p_{i,s}^{t}\\ & -\sum_{i=1}^{10}2G_{i}^{t+\Delta t}\left\{(1-\exp(-\frac{\Delta t}{\tau_{i}}))e_{ij}(t)+(\exp(-\frac{\Delta t}{\tau_{i}})-1)p_{i,s}^{t}\right\} \end{array}$$

where:

$$p_{i,s}^{t+\Delta t}=\int_{0}^{t+\Delta t}(1-\exp(-\frac{t+\Delta t-\tau }{\tau_{i}}))\frac{\partial e_{ij}}{\partial \tau }d\tau$$

The increment of the stress volumetric tensor is as follows:

(18) $$\begin{array}{ll} \Delta {\tilde{\sigma }}_{kk}\left(t+\Delta t\right) & =3K_{0}^{t+\Delta t}\Delta \varepsilon_{kk}-\sum_{i=1}^{10}3K_{i}^{t+\Delta t}\frac{\Delta \varepsilon_{kk}}{\Delta t}\left\{\Delta t-\tau_{i}(1-\exp(-\frac{\Delta t}{\tau_{i}}))\right\}\\ & +\Delta K_{0}^{t+\Delta t}\varepsilon_{kk}(t)-\sum_{i=1}^{10}3\Delta K_{i}^{t+\Delta t}p_{i,k}^{t}\\ & -\sum_{i=1}^{10}3K_{i}^{t+\Delta t}\left\{(1-\exp(-\frac{\Delta t}{\tau_{i}}))\varepsilon_{kk}(t)+(\exp(-\frac{\Delta t}{\tau_{i}})-1)p_{i,k}^{t}\right\} \end{array}$$

where:

$$p_{i,k}^{t+\Delta t}=\int_{0}^{t+\Delta t}(1-\exp(-\frac{t+\Delta t-\tau }{\tau_{i}}))\frac{\partial \varepsilon_{kk}}{\partial \tau }d\tau$$

The expression for the tangent stiffness matrix is as follows:

(19) $$D_{ijkl}\left(t+\Delta t\right)=\frac{\partial \Delta \sigma_{ij}\left(t+\Delta t\right)}{\partial \Delta \varepsilon_{kl}\left(t+\Delta t\right)}$$

Combining Equations (16)–(18), the tangent stiffness at time *t* + ∆*t* can be obtained as follows:

(20) $$D_{iiii}(t+\Delta t)=\frac{1}{3}(\frac{2}{1+\mu }+\frac{1}{1-2\mu })A_{1}$$

(21) $$D_{iijj}(t+\Delta t)=\frac{1}{3}(\frac{1}{1-2\mu }-\frac{1}{1+\mu })A_{1}\;\;(i\neq j)$$

(22) $$D_{ijij}(t+\Delta t)=\frac{1}{2(1+\mu )}A_{1}\;\;(i\neq j)$$

where:

$$A_{1}=E_{0}^{t+\Delta t}-\sum_{i=1}^{10}\frac{E_{i}^{t+\Delta t}}{\Delta t}(\Delta t-\tau_{i}(1-\exp(-\frac{\Delta t}{\tau_{i}}))).$$

Based on earlier constitutive relationships and the stress update method described here, the aging viscoelastic constitutive model has been further developed. The model was also refined using UMAT subroutine guidelines.

### 3.4. The Verification of the Model

**Figure 7:**
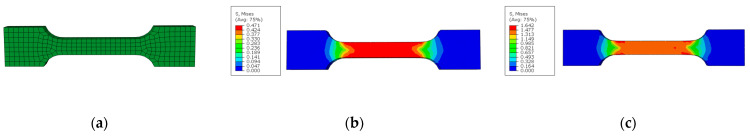
(**a**) Finite element model of the specimen; (**b**) stress distribution diagram of simulated tensile test; (**c**) stress distribution diagram of simulated relaxation test.

The aging viscoelastic constitutive model is verified by simulating the uniform stretching and relaxation processes of aged specimens. The finite element model (FEM) for the standard dumbbell-shaped specimen (Figure 1) is constructed using 3 mm hexahedral mesh elements. This model comprises 702 C3D8R elements, as shown in Figure 7a. The relaxation modulus parameters of the specimens are provided earlier in the text.

**Figure 8:**
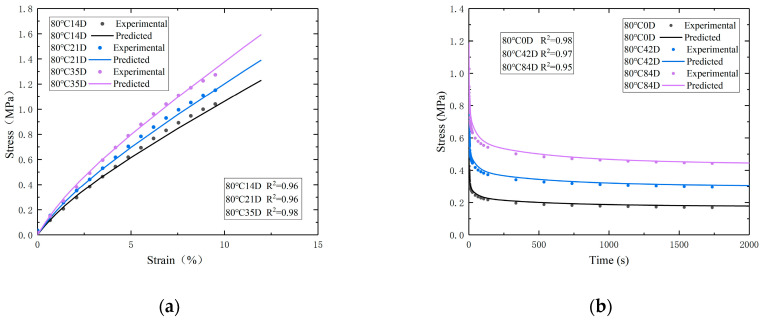
(**a**) The stress–strain curves of experimental/predicted tensile tests. (**b**) The stress–time curves of experimental/predicted relaxation tests.

The stress distribution from the tensile tests and the relaxation tests are shown in Figure 7b and Figure 7c, respectively. The relaxation curves and tensile curves were compared with the FEA results. The results are shown in Figure 8. Overall, the experimental curves align well with the predicted curves.

## 4. Estimation Method for the Storage Life of PFPCs

### 4.1. Safety Margin Assessment

For the failure analysis of PFPCs, the failure criterion based on equivalent strain is generally used [35]:

(23) $$\varepsilon <\varepsilon_{m}$$

where $\varepsilon_{m}$ represents the limit strain value, and *ε* represents the calculated strain of PFPCs. The material of the column studied in this paper is commonly used in aerospace applications. Therefore, to enhance the safety of the column, the safety factor was chosen as 1.8 [36,37].

In this study, the dewetting strain of PFPCs is used as the limit strain. When aging is ignored, the dewetting strain is 10.15%. From the experimental results, it can be observed that as the aging level increases, the dewetting strain gradually decreases.

Related research suggests that there is a statistical relationship between dewetting strain and crosslinking [24], as follows:

(24) $$\varepsilon_{m}=\alpha v^{\beta }$$

**Figure 9:**
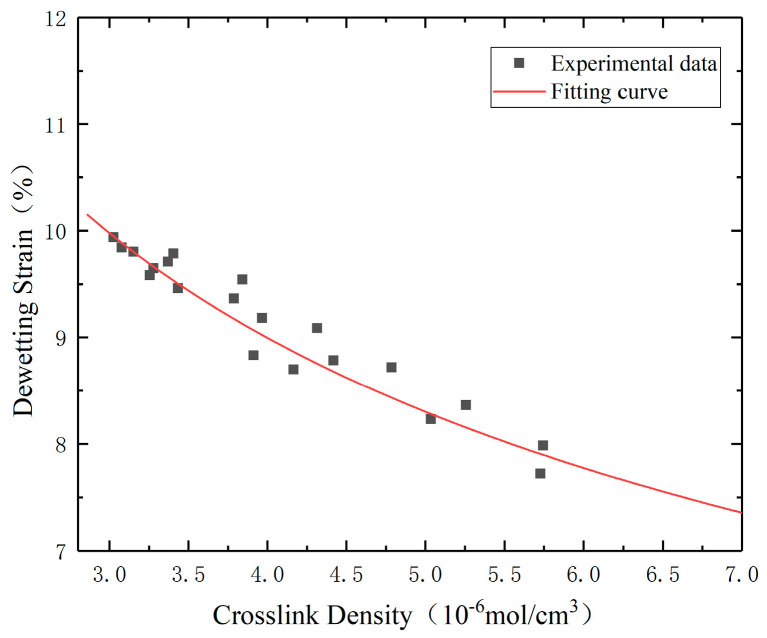
Diagram of crosslink density–dewetting strain fitting for the specimen.

The values of *α* and *β* can be obtained from the fitting results of the power function. The fitting results show *α =* 0.11 and *β =* −0.36 in Equation (24). According to calculations, the R^2^ value of the dewetting strain prediction model is 0.95, which indicates that the model has acceptable accuracy and reliability. The fitting results are shown in Figure 9.

### 4.2. Case Analysis of PFPC Column

This study focuses on a PFPC column for which a three-dimensional FEM is established, simplified to match practical conditions. It assumes the casing is tightly bonded, excluding the artificial debonding layers at both ends. The outer surface is bonded and fixed, whereas the inner bore surfaces remain free.

**Figure 10:**
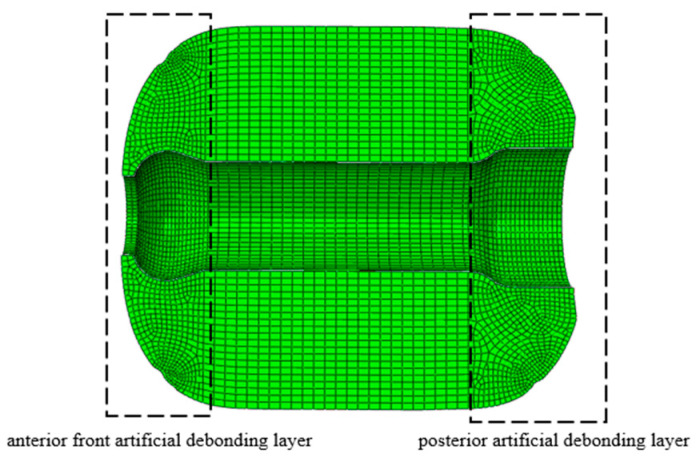
Finite element model of the column.

The column, made of a viscoelastic material, has a length of 2.3 m, an outer diameter of 1.8 m, and an inner diameter of 0.6 m. The FEM is shown in Figure 10, which consists of 80,470 C3D8R elements. To capture the detailed behavior near the artificially debonded layer, mesh refinement was applied, with element sizes ranging from 20 mm to 40 mm. The parameters corresponding to its constitutive model are the same as those mentioned earlier in the text. The density of the model is 1800 kg/m^3^, with a Poisson’s ratio of 0.48. During long-term storage, the environmental temperature has a significant impact on the aging of the PFPC. The environmental temperature is set to 25 °C for calculations. The column is stored horizontally in a warehouse, mainly affected by gravitational loads, and it is subject to an acceleration of 1 g in the *z*-axis direction.

**Figure 11:**
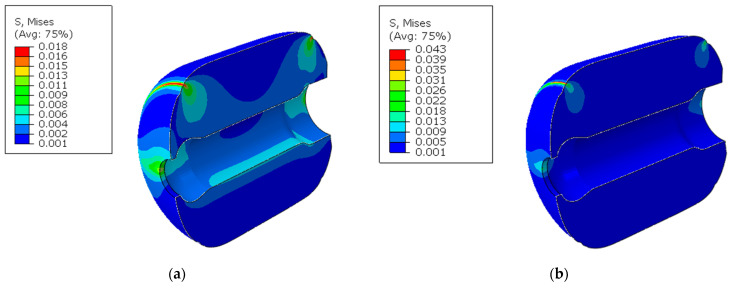
Von Mises stress distribution under gravitational load: (**a**) ignoring aging effects; (**b**) considering aging effects.

**Figure 12:**
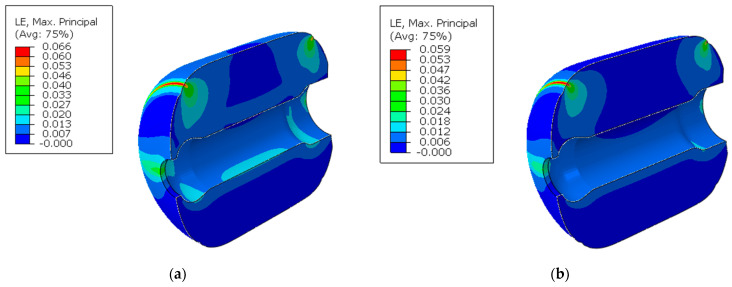
Strain distribution under gravitational load: (**a**) ignoring aging effects; (**b**) considering aging effects.

This section primarily simulates the structural response of a PFPC column under two scenarios: with and without aging effects. Through calculations, the stress and strain distribution after 25 years of storage at 25 °C can be obtained. The results are shown in Figure 11 and Figure 12.

When the aging effects are ignored, the stress distribution of the column is shown in Figure 11a, and the strain distribution is shown in Figure 12a. Since the ends of the cylinder are near the artificially debonded layer, no constraints are set at the upper part, resulting in a free boundary. Under the influence of gravity, the stress and strain near the artificially debonded layer are relatively large. Additionally, the inner wall of the cylinder is a free boundary, leading to increased stress and strain under gravity. Under the influence of gravitational load, the stress and strain are primarily concentrated at the artificial debonding layers at the front and back and the inner bore, respectively. Particularly at the root of the front artificial debonding layer at the top of the column, the stress reaches a maximum value of 0.018 MPa, and the strain peaks at 6.09%. When considering the aging effects, the stress and strain distribution are shown in Figure 11b and Figure 12b. Aging increases the relaxation modulus without changing the cylinder’s physical constraints, leading to higher stress and lower strain under the same load. Under the same gravitational load, the distributions of stress and strain are similar to those when aging effects are ignored due to unchanged gravitational load and boundary conditions. The stress at the root of the front artificial debonding layer remains the highest at 0.043 MPa, and the strain peaks at 5.93%.

A comparison reveals that after 25 years of storage, Ignoring aging effects results In lower stress but higher strain in the column than when considering them. This is because the aging viscoelastic constitutive model used in the calculations includes the aging effects, which account for the increased PFPC modulus. This increase reflects the hardening phenomenon caused by the aging crosslinking of the PFPC binder.

**Figure 13:**
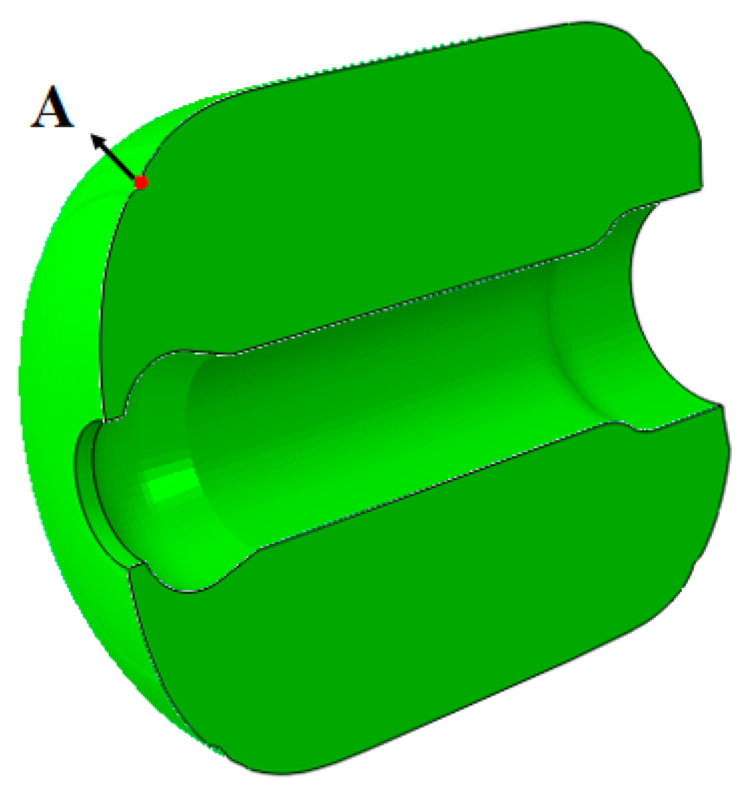
Location of high strain points in the model.

**Figure 14:**
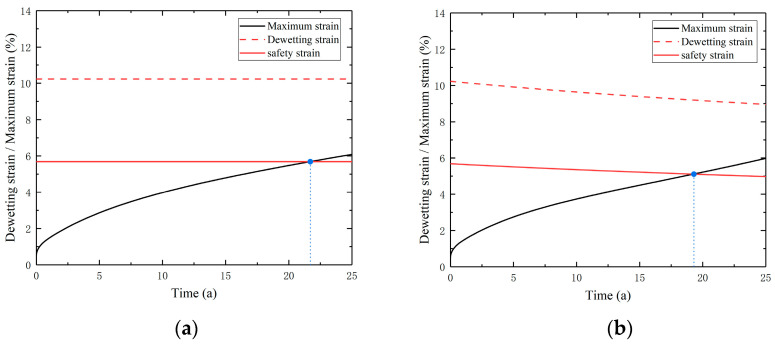
Failure criteria chart for horizontal storage of the model: (**a**) ignoring aging effects; (**b**) considering aging effects.

The root of the front artificial debonding layer is a critical area for life estimation. The maximum strain point A in this area is shown in Figure 13. By placing the maximum strain curve and the dewetting strain curve on the same time axis, the storage life of the column can be estimated. The failure criteria under both conditions, ignoring aging effects and considering aging effects, are shown in Figure 14. 

When the maximum strain of the columns reaches the dewetting strain indicative of failure, they are deemed to have failed. A safety factor of 1.8 is chosen by taking into consideration that a sufficient safety margin is needed in practical applications. Analysis of Figure 14 reveals that there is an intersection between the maximum strain and the dewetting strain, considering the safety factor. The time corresponding to this intersection is the storage life of the column, representing the maximum storage time under horizontal storage conditions. When the aging effects are ignored, the storage life is about 22 years, whereas the storage life is reduced to 19 years when considering aging effects. There is a significant difference in the calculated results of strain between ignoring and considering aging effects. The aging viscoelastic constitutive model reflects material hardening caused by aging, leading to a decrease in strain. A reduction in dewetting strain was also caused by aging. When the combined effects of aging on maximum strain and dewetting strain were considered, the estimated storage life of the column was shortened. The safety margin assessment method based on dewetting strain provides a reference for estimating the life expectancy of PFPCs while considering the aging effect. 

## 5. Conclusions

In this work, experimental and numerical studies were conducted to investigate the aging performance of PFPCs. An aging viscoelastic constitutive model was established, which integrates a safety margin assessment method based on dewetting strain, to predict the storage life of a PFPC column. Specific conclusions are as follows:

(1) The degree of aging of the PFPCs was characterized using crosslink density. Combined with the Arrhenius equation, a crosslink density prediction model was established. This model can be used to determine the crosslink density of samples at different aging stages. Then, based on the mathematical relationship between crosslink density and relaxation modulus, an aging model for the relaxation modulus was developed, as well as an aging viscoelastic constitutive model.
(2) Based on the aging relaxation modulus, an aging viscoelastic constitutive model was established. An integral algorithm was used for the numerical discretization of this constitutive model. The UMAT subroutine of ABAQUS was employed to compute the stress and strain behavior of the PFPC column in a horizontal storage condition. The results show that stress values are increased while strain values are reduced when considering aging effects. Compared to the constitutive model that ignores aging, the hardening effect of the PFPCs caused by aging can be characterized by the aging viscoelastic constitutive.
(3) Based on the maximum stress and the safety margin assessment method using dewetting strain, the PFPC column with a length of 2.3 m and outer diameter of 1.8 m storage life is estimated. The results show that when aging effects are ignored, the estimated storage life is 22 years, and when aging effects are considered, the estimated storage life is 19 years. The predicted storage life of the PFPC column is reduced when considering aging effects. This method provides a reference for predicting the storage life of PFPCs.

## Figures and Tables

**Table 1:** Table of accelerated aging test conditions.

Temperature (°C)	Aging Time (d)							
60	14	28	42	56	84	112	140	168
70	7	14	21	35	42	63	84	112
80	7	14	21	28	35	42	63	84

**Table 2:** Table of relaxation test conditions.

Temperature (°C)	Aging Time (d)	
60	42	84
70	42	84
80	42	84

**Table 3:** Table of relaxation modulus parameter data.

	1	2	3	4	5	6	7	8	9	10	∞
*E_i_*/MPa	7.2	2.11	1.43	0.98	0.41	0.19	0.71	0.85	0.93	0.28	0.13
*τ_i_*/s	0.5	5	5 × 10	5 × 10^2^	5 × 10^3^	5 × 10^4^	5 × 10^5^	5 × 10^6^	5 × 10^7^	5 × 10^8^	-

## Data Availability

The raw/processed data required to reproduce these findings cannot be shared at this time, as the data also form part of an ongoing study.
